# Supplementary material for: Complexome profiling of the Chlamydomonas psb28 mutant reveals TEF5 as an early PSII assembly factor
Source: Plant Cell. 2025 Mar 17;37(6):koaf055. doi: 10.1093/plcell/koaf055 (PMC12164590; doi:10.1093/plcell/koaf055)
Supplement: koaf055_Supplementary_Data [file koaf055_supplementary_data.zip › psb28_interactive.zip!/plots/intensity/qualityPlots/peptides/CPLD48_Cre11.g468750.t1.2.html]

### Cre11.g468750.t1.2

Trivial name: CPLD48  
SeqCoverage [median]: 28.00  
Mass: 24.81 kDa  
  
 

| Sequence | MissedCleavage | Mass [Da] | wtIntensity | muIntensity | Score |
| --- | --- | --- | --- | --- | --- |
| AIRQELETK | 1 | 1086.60 | 12298.08 | 25328.87 | 142.00 |
| AMGELVEAVK | 0 | 1045.55 | 2583.64 | 10405.62 | 151.44 |
| AMGELVEAVKK | 1 | 1173.64 | 24077.56 | 45275.36 | 133.07 |
| IKAIRQELETK | 2 | 1327.78 | 0.00 | 2254.19 | 57.35 |
| KLKPDGFTPK | 2 | 1129.65 | 0.00 | 1342.39 | 82.42 |
| QELETK | 0 | 746.38 | 1305.35 | 1433.41 | 126.20 |
| SASRVGESDGNINR | 1 | 1460.70 | 0.00 | 899.57 | 92.53 |
| THYAPPLTYNPEDGR | 0 | 1729.81 | 3336.33 | 17271.77 | 126.63 |
| THYAPPLTYNPEDGRGK | 1 | 1914.92 | 417.68 | 2352.16 | 76.39 |
| VGESDGNINR | 0 | 1059.49 | 22264.30 | 43791.04 | 201.21 |
| VGESDGNINRK | 1 | 1187.59 | 859.12 | 2386.34 | 79.20 |
|  |  |  | 67142.1 | 152740.7 |  |
